# Serum amyloid A augments the atherogenic effects of cholesteryl ester transfer protein

**DOI:** 10.1016/j.jlr.2023.100365

**Published:** 2023-03-31

**Authors:** Ailing Ji, Andrea C. Trumbauer, Victoria P. Noffsinger, Frederick C. de Beer, Nancy R. Webb, Lisa R. Tannock, Preetha Shridas

**Affiliations:** 1 Barnstable Brown Diabetes Center, University of Kentucky, Lexington, KY, USA; 2 Saha Cardiovascular Research Center, University of Kentucky, Lexington, KY, USA; 3 Department of Internal Medicine, University of Kentucky, Lexington, KY, USA; 4 Department of Pharmacology and Nutritional Sciences, University of Kentucky, Lexington, KY, USA; 5 Lexington Veterans Affairs Medical Center, Lexington, KY, USA

**Keywords:** serum amyloid A, cholesteryl ester transfer protein, atherosclerosis, lipid metabolism, apolipoprotein, HDL, inflammation

## Abstract

Serum amyloid A (SAA) is predictive of CVD in humans and causes atherosclerosis in mice. SAA has many proatherogenic effects in vitro. However, HDL, the major carrier of SAA in the circulation, masks these effects. The remodeling of HDL by cholesteryl ester transfer protein (CETP) liberates SAA restoring its proinflammatory activity. Here, we investigated whether deficiency of SAA suppresses the previously described proatherogenic effect of CETP. ApoE^−/−^ mice and apoE^−/−^ mice deficient in the three acute-phase isoforms of SAA (SAA1.1, SAA2.1, and SAA3; “apoE^−/−^ SAA-TKO”) with and without adeno-associated virus-mediated expression of CETP were studied. There was no effect of CETP expression or SAA genotype on plasma lipids or inflammatory markers. Atherosclerotic lesion area in the aortic arch of apoE^−/−^ mice was 5.9 ± 1.2%; CETP expression significantly increased atherosclerosis in apoE^−/−^ mice (13.1 ± 2.2%). However, atherosclerotic lesion area in the aortic arch of apoE^−/−^ SAA-TKO mice (5.1 ± 1.1%) was not significantly increased by CETP expression (6.2 ± 0.9%). The increased atherosclerosis in apoE^−/−^ mice expressing CETP was associated with markedly increased SAA immunostaining in aortic root sections. Thus, SAA augments the atherogenic effects of CETP, which suggests that inhibiting CETP may be of particular benefit in patients with high SAA.

Serum amyloid A (SAA) is a highly conserved family of acute-phase reactants that are thought to play an important role in innate immunity (1, 2). In response to an acute infection or tissue damage, SAA secretion by the liver increases transiently up to 1,000-fold and then returns to normal levels within a few days (2, 3). SAA is also chronically elevated, albeit at more modest levels, in individuals with chronic inflammatory conditions, including rheumatoid arthritis, obesity, and type 2 diabetes, which are all recognized to increase the risk for CVD (4, 5). Numerous epidemiological studies over the past several decades have identified a strong association between circulating SAA concentrations and all-cause and cardiovascular mortality, and there is a strong independent relationship in humans between SAA and future cardiovascular events (6, 7).

SAA has also been associated with atherosclerotic CVD in mouse models. For example, addition of cholesterol to a high-fat diet increases plasma SAA levels and atherosclerosis in LDL receptor-deficient (Ldlr^−/−^) mice (8). SAA is also significantly elevated in obese apoE^−/−^ mice with accelerated atherosclerosis (9). Many gain-of-function and loss-of-function studies in mice demonstrate that SAA plays a causal role and is not just a biomarker in the development of atherosclerosis. Overexpression of SAA using a murine lentivirus accelerates the progression of atherosclerosis in apoE^−/−^ mice (10). Even a transient increase in SAA mediated by adenoviral vector gene delivery results in significantly increased atherosclerosis in apoE^−/−^ mice (11). Conversely, deficiency of all acute-phase SAA isoforms leads to decreased atherosclerosis in apoE^−/−^ mice (12), and Ldlr^−/−^ mice lacking SAA1.1 and SAA2.1 demonstrate a reduction of early lesion formation in the ascending aorta (13).

In vitro studies have identified numerous potentially proatherogenic activities mediated by SAA. SAA is a chemoattractant for neutrophils and monocytes (14), induces the expression of various inflammatory cytokines, including interleukin 6 (IL6) and TNFα (15), and activates the NACHT, LRR, and PYD domain-containing protein 3 (NLRP3) inflammasome to stimulate IL-1β activation (16). These activities have been attributed to the ability of SAA to serve as a ligand for a number of pattern recognition receptors, including formyl peptide receptor-like 1 (17) and 2 (18), Toll-like receptors 2 (19) and 4 (20), and class B scavenger receptor BI (21). Notably, the majority of SAA in the circulation is HDL associated and not in a lipid-free form (22, 23), but only lipid-free SAA, and not HDL-associated SAA appears to be bioactive in vitro (14, 16, 24). Thus, HDL, the major carrier of SAA in the circulation, may serve to sequester and thereby neutralize the proinflammatory and proatherogenic effects of SAA in vivo, possibly to protect the host from systemic tissue damage during an acute phase response. Issues yet to be addressed are the mechanism(s) by which SAA is released from HDL to exert its functions during an innate immune response and whether the dissociation of SAA from HDL in the setting of chronic inflammation leads to proatherogenic effects.

Cholesteryl ester transfer protein (CETP) is a hydrophobic glycoprotein of hepatic origin that circulates in plasma bound mainly to HDL (25). It plays an important role in the metabolism of circulating lipoproteins. CETP promotes the transfer of CE from HDL to apoB-containing lipoprotein particles in exchange for triglyceride (TG) and consequently, mediates the clearance of HDL-derived CE by LDL receptors in the liver (26). CETP remodeling of HDL results in an increase of lipid-poor apoA-I (27, 28). We reported that CETP remodeling of HDL also liberates lipid-free SAA and leads to the transfer of SAA from HDL to LDL/VLDL (23). Whether CETP-mediated remodeling of SAA-enriched HDL impacts the biological activities of SAA in vitro and in vivo has not been investigated. Mice normally lack CETP, and the expression of human CETP in transgenic mice results in moderate increases in atherosclerosis in both apoE^−/−^ and Ldlr^−/−^ mice (29). In the current study, we tested the hypothesis that the presence of SAA contributes to the proatherogenic effect of CETP in mice.

## Materials and methods

### Animals

ApoE^−/−^ mice on a C57BL/6 background were obtained from the Jackson Laboratory. Mice deficient in SAA1.1, SAA2.1, and SAA3 (SAA1.1/2.1/3-TKO) were generously provided by Drs. June-Yong Lee and Dan Littman, New York University. The SAA1.1/2.1/3-TKO mice were generated by inserting a premature stop codon into exon 2 of *saa3* in the SAA1.1/2.1-DKO mouse using CRISPR-Cas9 technology as described previously (12, 30). The SAA1.1/2.1/3-TKO mice were then bred to apoE^−/−^ mice to generate apoE^−/−^ mice lacking all acute-phase SAA isoforms (apoE^−/−^ SAA-TKO). The apoE^−/−^ and apoE^−/−^ SAA-TKO mice from this cross were then bred in parallel, and age-matched and littermate apoE^−/−^ and apoE^−/−^ SAA-TKO mice were used in the atherosclerosis study. Two batches of adeno-associated virus (AAV; serotype 8) expressing human CETP (AAV8-TBG-h-CETP) were used in this experiment. Pilot studies were performed to determine dosing based on equivalent plasma CETP activities between batches. The first batch was kindly provided by Dr. Graf at the University of Kentucky, and the second batch was provided by Vector Biolabs (Malvern, PA). Empty AAV vector (AAV-null) was used as the control. Atherosclerotic lipid accumulation was assessed in male apoE^−/−^ and apoE^−/−^ SAA-TKO mice (13 weeks old at the start of the experiment) injected i.p. with 3 × 10^10^ and 1 × 10^11^ particles of AAV-CETP and AAV-null for the first and second batch of experiments, respectively. Mice that do not show any detectable CETP activity when assayed 9 weeks after AAV injection were removed from the study (5/20 and 2/16 mice from apoE^−/−^ and apoE^−/−^ SAA TKO, respectively). All mice were fed a normal rodent diet and euthanized 14 weeks after AAV injection. Animals were housed in microisolator cages and maintained on a 14 h light/10 h dark cycle. Mice were provided with normal chow diet and water ad libitum. SAA transgenic mice (used for preparing HDL containing SAA—termed SAA-HDL) harboring an *Saa* transgene regulated by a tetracycline-responsive promoter were generously provided by Dr. Paul Simon (University College London) (31). All studies were performed in accordance with the Public Health Service Policy on Humane Care and Use of Laboratory Animals and with the approval of the University of Kentucky and Lexington Veterans Affairs Medical Center Institutional Animal Care and Use Committees.

### HDL preparation

Mouse and human HDLs (*d* = 1.063–1.21 g/ml) were isolated from C57BL/6 mice, TKO mice, SAA transgenic mice, and healthy human volunteers by density gradient ultracentrifugation as described previously (32). The human plasma was collected under an institutional board-approved protocol. HDL was dialyzed against 150 mM NaCl and 0.01% EDTA, sterile filtered, and stored under argon gas at 4°C. Protein concentrations were determined by the method of Lowry *et al.* (33). HDL purity and integrity were confirmed by SDS-PAGE (4–20% polyacrylamide SDS gels; Bio-Rad Laboratories, Hercules, CA) and nondenaturing gradient gel electrophoresis, in which HDL was electrophoresed on a 4–20% nondenaturing polyacrylamide gel for 3.5 h at 200 V, 4°C.

### CETP remodeling of HDL particles in vitro and macrophage activation assay

HDLs (0.2 mg HDL protein) prepared from TKO mice (TKO-HDL) and SAA transgenic mice (SAA-HDL) were incubated in a final reaction volume of 180 μl in Tris-buffered saline, pH 7.4, at 37°C for 24 h with recombinant human CETP (Cardiovascular Targets, Inc., NY; 60 μg/ml, 10.8 μg CETP), human VLDL (Millipore, catalog number: LP1; 4 mM final TG concentration), fatty acid-free BSA (10 mg/ml) and CaCl_2_ (2 mM) as published earlier (27). As a control, SAA-HDL was incubated with the remodeling cocktail under the same reaction conditions but without the addition of CETP. The reactions were terminated by the addition of EDTA (20 mM final concentration). The reaction mixtures were then desalted, concentrated using Amicon centrifugal filters (3K), washed and resuspended in serum-free DMEM media to final volume of 300 μl and directly added to confluent J774 cells plated 48 h before treatment. The conditioned media from the treated cells was collected after 24 h for IL-1β quantification by ELISA (R&D).

### RNA isolation and quantitative RT-PCR

Total RNA was isolated from liver according to the manufacturer's instructions (RNeasy® Mini Kit, Qiagen). RNA samples were incubated with DNase I (Qiagen) for 15 min at room temperature prior to reverse transcription. Liver RNA (1 μg) was reverse transcribed into complementary DNA using the Reverse Transcription System (Applied Biosystems). After 4-fold dilution, 5 μl was used as a template for real-time RT-PCR. Amplification was done for 40 cycles using Power SYBR Green PCR master mix kit (Applied Biosystems). Quantification of mRNA was performed using the ΔΔ*C*_*T*_ method and normalized to GAPDH. Primer sequences are as follows: GAPDH (NM_008084), 5′-CTC ATG ACC ACA GTC CAT GCC A-3′, 5′-GGA TGA CCT TGC CCA CAG CCT T-3′; SAA1.1 (NM_011314), 5′- GCC ATG GAG GGT TTT TTT CAT TTA TTG-3′, 5′-TTG TCT CCA TCT TTC CAG CC-3′; SAA2.1 (NM_009117), 5′-GCC ATG GAG GGT TTT TTT CAT TTA TTG-3′, 5′-GAG CAT GGA AGT ATT TGT CTG AG-3′.

### CETP, SAA, and IL-1β measurements

CETP activity levels in plasma were determined by measuring the transfer of neutral lipids from a synthetic substrate to a physiological acceptor using the CETP Activity Assay Kit (Sigma-Aldrich). Plasma SAA concentrations were determined using a mouse ELISA kit (catalog no.: TP802M; Tridelta Development Ltd, Ireland). Concentrations of IL-1β in plasma and cell culture media were determined using a mouse IL-1β ELISA kit (R&D Systems). For J774 cells, IL-1β in conditioned media was normalized to total cell protein to account for potential differences in seeding density.

### Plasma lipids and lipoprotein analysis

Plasma total cholesterol and TG were determined colorimetrically (Wako Chemicals). Lipoproteins in 50 μl of plasma pooled from five mice in each group were separated by fast performance liquid chromatography utilizing a Superose 6 column (catalog no.: 17-5172-01; GE Healthcare, Uppsala, Sweden). The relative cholesterol content of 0.5 ml fractions was determined using a commercially available kit (Total Cholesterol E kit from Wako, Richmond, VA).

### Immunohistochemistry

For immunohistochemical analysis of aortic root sections, tissues that were frozen in OCT compound (Tissue-Tek, Torrance, CA) were serially cut in 8 μm thick sections, covering a length of approximately 640 μm from the aortic sinus (where the aortic valve leaflets appear) to the distal region of the root. Sections were mounted on glass slides and fixed in 4% paraformaldehyde for 30 min and treated with 0.1% Triton X-100 in PBS for 15 min. After blocking in 1% BSA/PBS at room temperature for 2 h, slides were incubated overnight at 4°C with a combination of rabbit anti-mouse SAA (catalog no.: ab199030; Abcam, Cambridge, MA) and rat anti-mouse CD68 (catalog no.: ab53444; Abcam, Cambridge, MA) diluted 1:200 in 1% BSA/PBS for each primary antibody. After washes with PBS, SAA was detected using Alexa Fluor 488-labeled goat anti-rabbit IgG (1:200 dilution; Molecular Probes; Cambridge, MA), and CD68 was detected using Alexa Fluor 568-labeled goat anti-rat IgG (1:200 dilution; Molecular Probes). Slides were mounted using fluorescence-protecting medium containing 4′,6-diamidino-2-phenylindole (Vectashield; Vector Laboratories). Images were captured by fluorescence microscopy (Nikon Eclipse 80i microscope, Nikon Instruments), and areas of immunopositive staining were quantified using Nikon NIS-elements software.

### Quantification of atherosclerotic lesions

Atherosclerosis quantification was performed as recommended in a Scientific Statement from the American Heart Association (34). For en face analyses, the aorta was cut from the heart at the aortic root, the entire aorta was cleaned of adventitial tissue, longitudinally cut, and tissues were pinned onto black wax to expose intimal surfaces. Aortas were visualized using a dissecting microscope, which was equipped with a Nikon digital camera that captured an image directly into an analysis program. Aortic arches were defined as the region from the ascending arch to 3 mm distal to the subclavian artery. Areas of intima covered by atherosclerosis were delineated by two independent researchers who were blinded to the study and quantified using Nikon NIS-elements software. The lesion area was expressed as the percent of the total aortic arch surface area.

For analysis of lesion areas in aortic roots, tissues that were frozen in OCT were serially cut in 8 μm thick sections and mounted on glass slides as described previously. Sections were fixed with 4% paraformaldehyde and stained with Oil Red O. Oil Red O was prepared by diluting saturated stock solution (0.5% in isopropyl alcohol) with two thirds of volume of dH_2_O and filtering with 0.2 micron syringe filter. Sections were stained with Oil Red O for 10 min, washed with 60% isopropyl alcohol, and stained with hematoxylin for 10 min to visualize nuclei. Atherosclerotic lesion area was delineated visually using Oil Red O staining and quantified by two independent researchers who were blinded to the study groups using Nikon NIS-elements software.

### Statistical analysis

Results are expressed as the mean ± SEM as indicated in the figure legends. Statistics were calculated with GraphPad’s Prism 9 software (GraphPad Software, Inc.). For experiments containing two groups, results were analyzed by Student's *t*-test. Plasma SAA levels over time by treatment were analyzed using two-way ANOVA followed by Tukey’s multiple comparisons test. The effects of CETP, SAA genotype, and their interaction on lesion area in the aortic root region and the percent of the total aortic arch surface area were analyzed using two-way ANOVA followed by Tukey’s multiple comparisons test. *P* values less than 0.05 were considered statistically significant and denoted in figures with a single asterisk (∗); *P* values less than 0.01 and 0.001 were denoted with two (∗∗) and three asterisks (∗∗∗), respectively.

## Results

### SAA-enriched HDL remodeled with CETP activates the NLRP3 inflammasome in macrophages

**Fig. 1:**
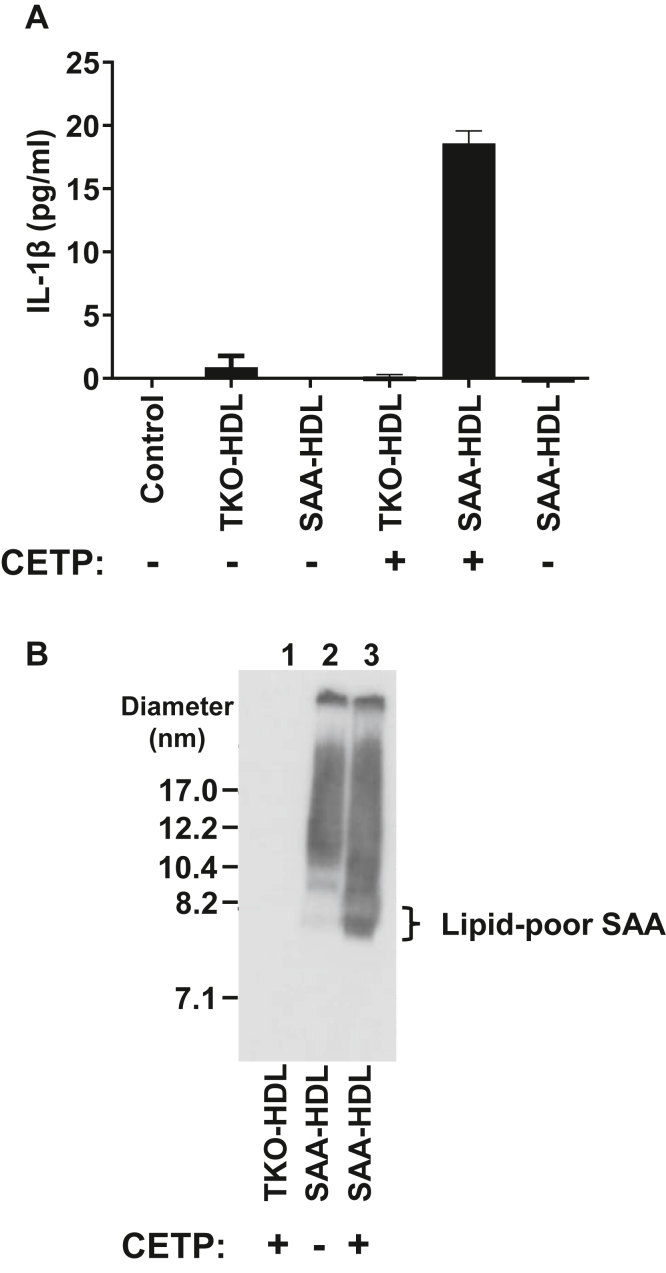
CETP remodeling of SAA-enriched HDL triggers IL-1β secretion in macrophages. A: J774 cells were incubated for 24 h with serum-free media lacking HDL (“control”) or serum-free media containing TKO-HDL (1.18 mg devoid of SAA) or SAA-HDL (1.18 mg HDL protein containing 0.19 mg of SAA) with or without CETP remodeling as indicated. IL-1β levels in the conditioned media were quantified by ELISA. B: TKO-HDL remodeled with CETP (lane 1), SAA-HDL treated with remodeling mixture minus CETP (lane 2), and SAA-HDL incubated with remodeling mixture containing CETP (lane 3) were separated by NDGGE followed by Western blotting to detect SAA. About 5 μl of the reaction mix was loaded per well.

We previously reported that lipid-poor SAA, but not HDL-bound SAA, activates the NLRP3 inflammasome in macrophages (16). We have also shown that CETP-mediated remodeling of SAA-enriched HDL leads to the liberation of lipid-poor SAA (23). Accordingly, it was of interest to investigate whether remodeling of SAA-enriched HDL by CETP unmasks the protective effect of HDL. J774 cells were incubated with media containing HDL from SAA transgenic mice (SAA-HDL), SAA-deficient mice (TKO-HDL), or SAA-HDL or TKO-HDL remodeled with CETP (TKO-HDL + CETP or SAA-HDL + CETP, respectively). As expected, both SAA-HDL and TKO-HDL failed to activate macrophages to release IL-1β (Fig. 1Α). CETP remodeling of SAA-HDL, but not TKO-HDL (lacking SAA), triggered IL-1β release in J774 cells. The ability of SAA-HDL to stimulate IL-1β release was dependent on CETP, since SAA-HDL treated with the remodeling cocktail without CETP (SAA-HDL − CETP) was inactive (Fig. 1A). Analysis of HDLs by nondenaturing gradient gel electrophoresis followed by Western blotting for SAA indicated that incubating SAA-HDL with CETP resulted in the generation of lipid-poor SAA (Fig. 1B, compare lanes 2 and 3).

### The effect of CETP on atherosclerosis depends, at least in part, on the presence of SAA

**Fig. 2:**
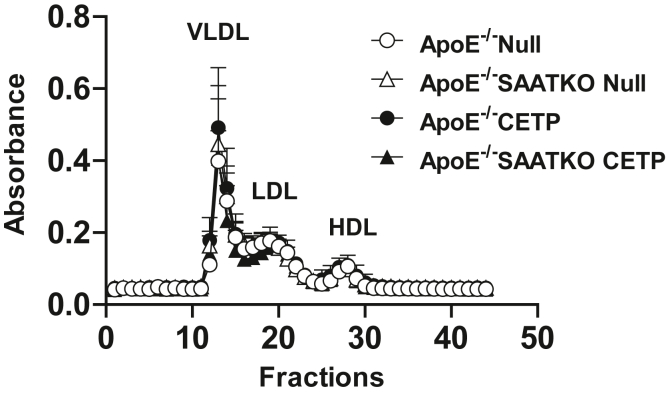
Expression of CETP or lack of SAA does not change lipoprotein profiles. Mice deficient in apoE (apoE^−/−^) and apoE^−/−^ mice deficient in all three inducible SAA isoforms (apoE^−/−^ SAATKO) were administered an AAV-expressing CETP or a null AAV and fed a normal chow diet for 14 weeks. Plasma was collected at the end of the study and fractionated by fast performance liquid chromatography; cholesterol content of 0.5 ml fractions was determined enzymatically (Wako Chemicals). apoE^−/−^ null (n = 5), apoE^−/−^ SAATKO null (n = 5), apoE^−/−^ CETP (n = 7), and apoE^−/−^ SAATKO CETP (n = 7).

**Fig. 3:**
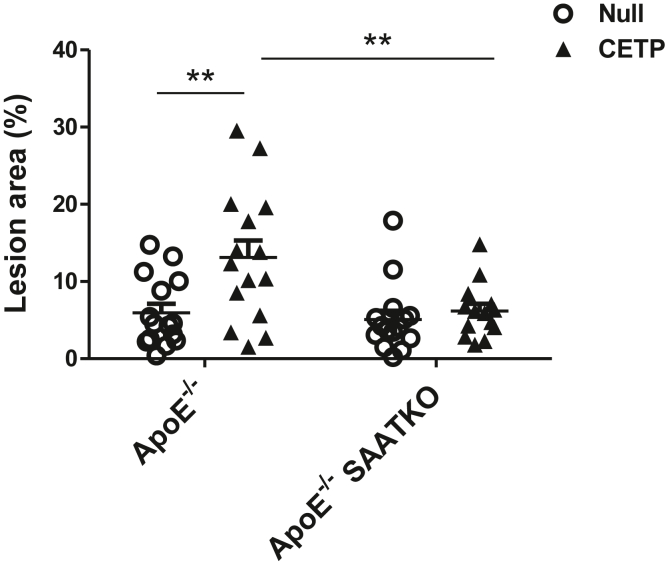
CETP expression increases atherosclerosis only in apoE^−/−^ mice and not in apoE^−/−^ SAATKO mice. ApoE^−/−^ and apoE^−/−^ SAATKO mice were injected with an AAV-expressing CETP or a null AAV and fed a normal chow diet for 14 weeks. Atherosclerosis in the aortic arch region was quantified by en face analysis. Each point represents an individual mouse with mean ± SEM shown for the group; expressed as the percent of the total aortic arch surface area. Statistical analysis was performed using two-way ANOVA followed by Tukey’s multiple comparisons test. apoE^−/−^ null (n = 15), apoE^−/−^ SAATKO null (n = 15), apoE^−/−^ CETP (n = 15), and apoE^−/−^ SAATKO CETP (n = 14).

To investigate whether the proatherogenic properties of CETP are altered by the presence or the absence of SAA, apoE^−/−^ and apoE^−/−^ SAA-TKO mice were injected with AAV-CETP or AAV-null and fed a normal chow diet for 14 weeks. Plasma IL6, IL-1β, total cholesterol, and TG levels were similar in the four groups of mice at the end of the study (Table 1). Moreover, lipoprotein profiles were similar for the four groups (Fig. 2). Atherosclerotic lesions on the intimal surface of the aortic arch were quantified at the end of the study and expressed as the percent of the total aortic arch surface area. As expected, the expression of CETP increased atherosclerosis in apoE^−/−^ mice (13.1 ± 2.2% compared with 5.9 ± 1.2% for ApoE^−/−^ mice in the presence and absence of CETP expression, respectively; *P* = 0.005), confirming results from an earlier study (29). However, the expression of CETP did not affect atherosclerosis in apoE^−/−^ SAA-TKO mice (6.2 ± 0.9% compared with 5.1 ± 1.1% in the presence and absence of CETP expression, respectively, *P* = NS) (Fig. 3), and the interaction was significant (*P* = 0.043). Lesion area in aortic roots was also analyzed: atherosclerotic lesion area in the roots of apoE^−/−^ mice was 100,405 ± 27,024 μm^2^, and CETP expression significantly increased atherosclerosis in apoE^−/−^ mice (416,848 ± 80,959 μm^2^) (supplemental Fig. S1A, B). Atherosclerotic lesion area in the roots of apoE^−/−^ SAA-TKO mice (22,501 ± 8,581 μm^2^) was not significantly enhanced by the expression of CETP (181,357 ± 77,950 μm^2^) (supplemental Fig. S1A, B). Thus, the proatherogenic effect of CETP in apoE^−/−^ mice is mediated, at least in part, via SAA, possibly because of the action of CETP to liberate SAA from HDL.

**Table 1:** Plasma cytokines and lipids levels and hepatic SAA mRNA levels

Cytokines/lipids/SAA mRNA	ApoE^−/−^ null (n = 5)	ApoE^−/−^ SAATKO null (n = 5)	ApoE^−/−^ CETP (n = 7)	ApoE^−/−^ SAATKO CETP (n = 7)	*P*
IL6 (pg/ml)	19.9 ± 8.5	6.6 ± 4.4	7.8 ± 4.3	22.5 ± 10.0	NS
IL-1β (pg/ml)	0.0 ± 0.0	0.6 ± 0.6	6.1 ± 4.1	6.2 ± 3.6	NS
Total cholesterol (mg/dl)	412.3 ± 44.9	486.7 ± 20.2	456.5 ± 53.8	374.3 ± 52.9	NS
TG (mg/dl)	143.9 ± 23.6	179.2 ± 15.1	143.1 ± 22.0	95.1 ± 6.6	NS
SAA1.1 (RE)	7.5 ± 2.7	—	0.5 ± 0.2	—	<0.05
SAA2.1 (RE)	5.2 ± 1.3	—	0.4 ± 0.2	—	<0.01

Mice deficient in apoE (apoE^−/−^) and apoE^−/−^ in all three inducible SAA isoforms (apoE^−/−^ SAATKO) were administered an AAV-expressing CETP or a null AAV and fed a normal chow diet for 14 weeks. Cytokines and lipids levels in the plasma collected at the end of the study were determined, and statistical analysis was performed using one-way ANOVA. Data shown are mean ± SEM. At the end of the study, hepatic SAA mRNA expression was determined by real-time PCR, and statistical analysis was performed using two-tailed *t*-tests.

Abbreviations: NS, not significant; RE, relative expression.

### Expression of CETP in mice decreases plasma SAA levels

**Fig. 4:**
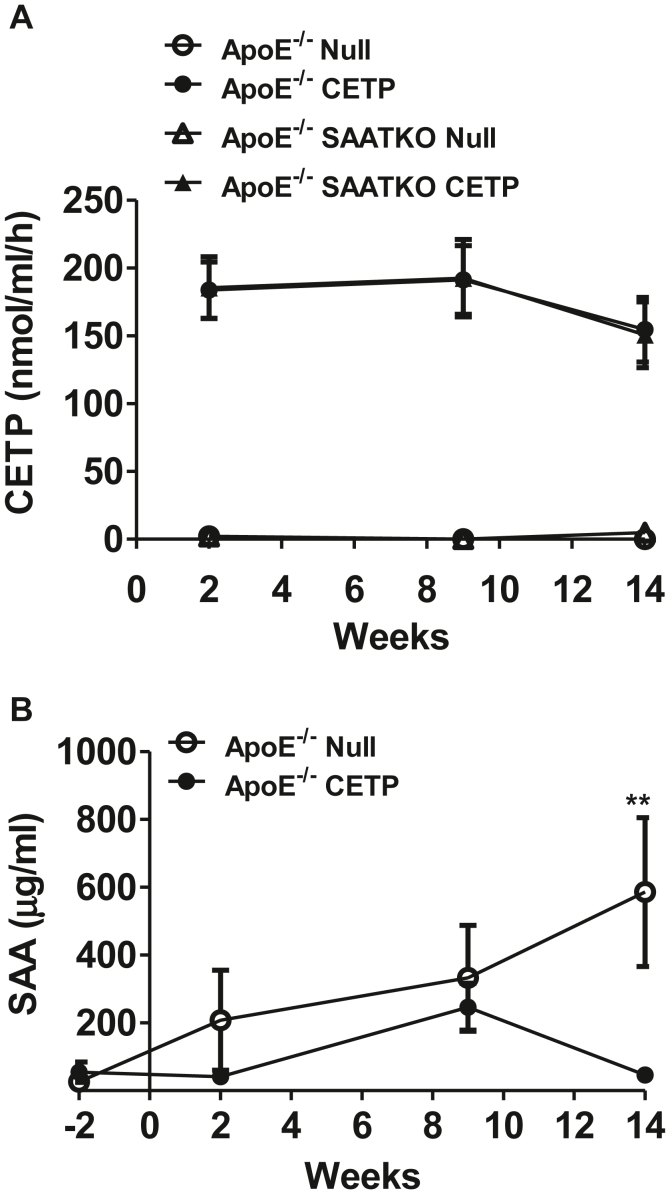
Expression of CETP in mice decreases plasma SAA concentrations. Mice deficient in apoE (apoE^−/−^) and apoE^−/−^ in all three inducible SAA isoforms (apoE^−/−^ SAATKO) were administered an AAV-expressing CETP or a null AAV and fed a normal chow diet for 14 weeks. A: CETP activity levels in plasma after 2, 9, and 14 weeks after AAV injection, determined by the detection of CETP-mediated transfer of neutral lipids from a synthetic substrate to a physiological acceptor. apoE^−/−^ null (n = 11), apoE^−/−^ SAATKO null (n = 11), apoE^−/−^ CETP (n = 15), and apoE^−/−^ SAATKO CETP (n = 16). B: SAA levels in the plasma collected at −2, 2, 9, and 14 weeks after AAV injection, determined by ELISA. Statistical analysis was performed using two-way ANOVA followed by Tukey’s multiple comparisons test: ∗∗*P* < 0.01, apoE^−/−^ null (n = 5), apoE^−/−^ CETP (n = 7).

We investigated the possibility that SAA augments the proatherogenic effect of CETP by modulating circulating CETP activity. CETP activity was assessed in all mice 2, 9, and 14 weeks after AAV-CETP or AAV-null injection. As expected, CETP was not detected in plasma of mice injected with AAV-null, as mice lack CETP (35). Two weeks after the administration of AAV-CETP, the CETP activity level increased to 195.4 ± 19.16 nmol/ml/h and remained elevated over 9 weeks, then decreased to approximately 155.8 ± 16.97 nmol/ml/h at 14 weeks. Notably, CETP activities were not significantly different between apoE^−/−^ mice and apoE^−/−^ SAA-TKO mice treated with AAV-CETP (Fig. 4A). To investigate whether CETP expression alters circulating SAA, plasma SAA levels 2, 9, and 14 weeks after AAV injection were determined. As shown in Fig. 4B, SAA levels in CETP-expressing mice were generally lower compared with mice lacking CETP throughout the course of the study, indicating that CETP does not enhance atherosclerosis development by increasing plasma SAA levels. The interaction between treatment and week was statistically significant (*P* = 0.03) suggesting that the effect of CETP on SAA levels differed by week. The most notable difference in mean SAA values appeared to be at week 14, where the mean SAA level in AAV-null-treated mice (586.0 μg/ml) was more than 10 times that of SAA-CETP-treated mice (46.4 μg/ml). The effect of CETP to lower plasma SAA levels was due at least in part to a reduction in hepatic expression of the major mouse acute phase SAAs, SAA1.1 and SAA2.1, in CETP-expressing mice (Table 1). While the mechanism of this effect is not known, our results are consistent with an earlier report showing increased hepatic SAA expression in CETP transgenic mice treated with a CETP inhibitor, anacetrapib (36). Furthermore, our data rule out the possibility that CETP enhances atherosclerosis by increasing circulating SAA concentrations.

### SAA is present in lesions of apoE^−/−^ mice expressing CETP

**Fig. 5:**
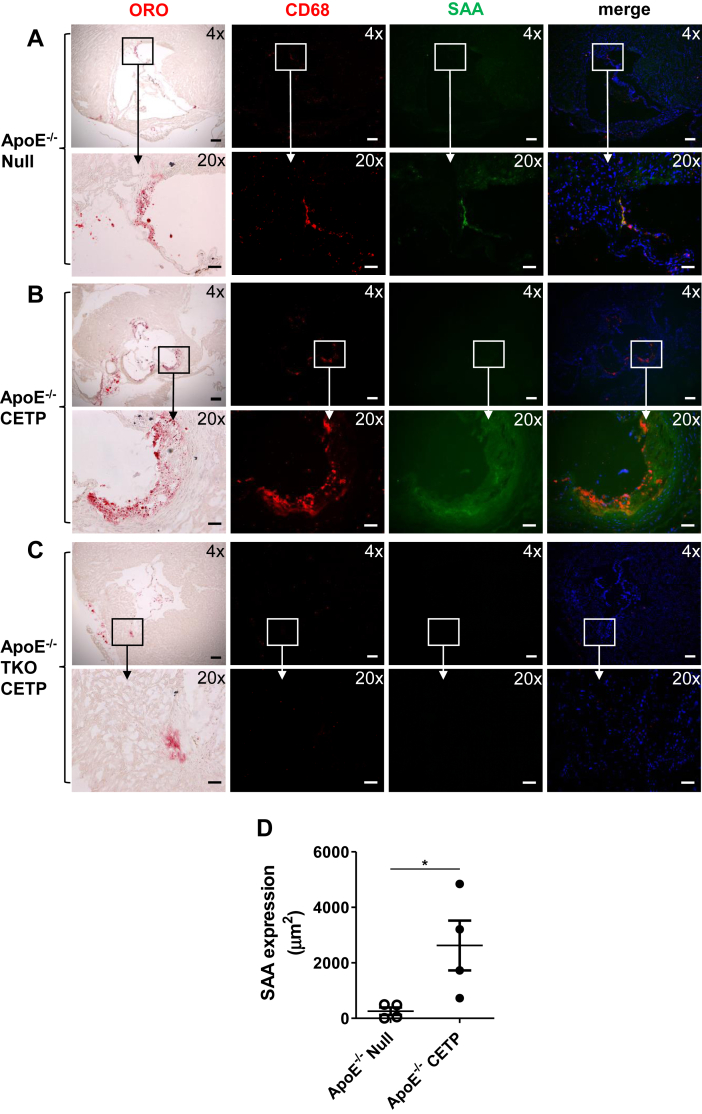
SAA is enhanced in the lesions of aortic roots of apoE^−/−^ mice expressing CETP. ApoE^−/−^ and apoE^−/−^ SAATKO mice were injected with an AAV-expressing CETP or a null AAV and fed a normal chow diet for 14 weeks. Aortic root sections from (A) apoE^−/−^ null mice, (B) apoE^−/−^ CETP mice, and (C) apoE^−/−^ TKO CETP mice were processed to detect macrophages (red fluorescence) and SAA (green fluorescence) by immunostaining, nuclei were identified using 4′,6-diamidino-2-phenylindole (blue fluorescence). Sections were photographed under 4× objective magnification, scale bar represents 200 μm and 20× objective magnification, scale bar represents 50 μm. D: SAA immunostaining in aortic roots of apoE^−/−^ null mice and apoE^−/−^ CETP mice was quantified using NIS-Elements software. Each point represents the area of SAA expression in the aortic root section of an individual mouse. Statistical analysis was performed using two-tailed *t*-tests, ∗*P* < 0.05.

We performed immunohistochemistry to investigate whether CETP can influence the amount of SAA that is present in the vessel wall, where it could have local proinflammatory and proatherogenic effects. Sections spanning the aortic roots of apoE^−/−^ mice with and without CETP expression were immunostained for CD68 to detect macrophages and SAA. Sections of the aortic roots from apoE^−/−^ TKO mice were used as a negative control for SAA immunoreactivity. As noted in Fig. 5, mice injected with AAV-CETP (Fig. 5B) had increased atherosclerotic lesion area compared with AAV-null (Fig. 5A). Prominent macrophage (red staining) and SAA (green staining) immunoreactivity was found in regions with extensive atherosclerosis in aortic roots of mice expressing CETP (Fig. 5B), with minimal staining in mice lacking CETP (Fig. 5A). As expected, SAA immunoreactivity was not detected in aortic roots of apoE^−/−^ TKO mice expressing CETP (Fig. 5C). The SAA was not colocalized to macrophage cells (Fig. 5B). Quantification of SAA immunoreactivity demonstrated increased SAA in lesions of aortic roots of apoE^−/−^ mice expressing CETP compared with control apoE^−/−^ mice (Fig. 5D). These results suggest that CETP expression in apoE^−/−^ mice leads to an increase in the amount of SAA in the vessel wall, despite decreased hepatic expression and plasma levels of SAA compared with apoE^−/−^ mice that lack CETP.

## Discussion

Elevated SAA is predictive of CVD events in numerous clinical studies (4, 5, 6, 7). We and others previously demonstrated that increased expression of SAA in mice leads to increased atherosclerosis (10, 11) and that suppression/deficiency of SAA limits atherosclerosis development (12, 37). However, SAA is predominantly found on HDL in vivo, and we and others have demonstrated that HDL-associated SAA is less proinflammatory compared with lipid-free SAA (14, 16, 24). Thus, how SAA actually exerts its proatherogenic effects in vivo has been unclear. We previously demonstrated that the remodeling of HDL-containing SAA by CETP leads to the transfer of SAA to apoB-containing lipoproteins and the liberation of small amounts of lipid-poor SAA (23, 27). Thus, we hypothesized that the proatherogenic effects of CETP may at least partially be attributed to its ability to liberate SAA from HDL. Consistent with this concept, in the present study, we demonstrated that SAA-containing HDL, but not SAA-free HDL, remodeled with CETP activates the NLRP3 inflammasome. We also determined that the deficiency of SAA impairs the proatherogenic effect of CETP in apoE^−/−^ mice.

By promoting the transfer of cholesteryl esters (CEs) from HDL to VLDL/LDL in exchange for TGs, CETP mediates the clearance of HDL-derived CE by hepatic LDL receptors, indicating an important role for CETP in reverse cholesterol transport (38). An additional consequence of CETP action is the generation of lipid-poor apoA-I from reconstituted HDL (28) and from acute phase HDL that is enriched with SAA (27). CETP-mediated remodeling of acute-phase HDL also leads to the release of lipid-poor SAA (23), which we propose serves as a mechanism for regulating the biological effects of SAA. Under normal conditions, almost all circulating SAAs are found in the HDL fraction, and it is now recognized that the activities of SAA are suppressed when it is associated with HDL (14, 16, 24, 39). For example, we previously reported that purified SAA stimulates IL-1β secretion in murine J774 macrophages through a mechanism that depends on NLRP3 expression and caspase-1 activity. However, incorporating SAA into HDL prior to its use in cell treatments completely eliminated its ability to stimulate IL-1β secretion (16), demonstrating a role for HDL in sequestering and neutralizing SAA, thus limiting inflammation. In the current study, we show for the first time that this remodeling “unmasks” the proinflammatory effects of HDL-associated SAA, as evidenced by the ability of the remodeled HDL to stimulate IL-1β secretion in vitro. The effect of CETP was clearly SAA dependent, since remodeling of normal HDL lacking SAA did not stimulate IL-1β secretion.

The impact of CETP on atherosclerosis has been investigated for several decades (38). CETP appears to have both proatherogenic and antiatherogenic properties. CETP can be considered as an accelerator of cholesterol flux through the reverse cholesterol transport pathway by causing HDL-derived CE to be transferred to the liver through receptor-mediated uptake of apoB-containing lipoproteins (38). However, this process leads to reduced HDL cholesterol levels, which can be considered to be proatherogenic. In an earlier study, transgenic expression of human CETP in 2- or 4-month-old apoE^−/−^ mice (which naturally lack CETP) resulted in a moderate ∼2-fold increase in atherosclerosis (29). The authors suggested that this increase in atherosclerosis may be due to the increased levels of VLDL and IDL cholesterol, reduced levels of HDL cholesterol, and impaired reverse cholesterol transport that was observed in the CETP transgenic mice. In our study, there was no significant difference in plasma cholesterol or lipoprotein cholesterol distribution in CETP-expressing mice compared with control. It is possible that these discrepant findings are due to differences in the mode of delivery of the human *CETP* gene, which resulted in differences in the duration and/or level of CETP expression, since our study utilized an AAV vector that induced maximal CETP expression beginning ∼6 weeks of age, rather than lifelong CETP expression in the previous study. However, despite the apparent lack of alterations in plasma lipoproteins in our study, CETP expression nevertheless led to significantly increased atherosclerosis in the aortic arch regions and aortic roots of apoE^−/−^ mice. Our data that the CETP-mediated increase in atherosclerosis is absent in mice lacking acute-phase SAAs provide novel insights into SAA biology and a possible mechanism for the proatherogenic effects of CETP. One possible limitation of our study was the use of the apoE^−/−^ model, which may conceal a potential role of SAA in regulating CETP-mediated CE flux to apoB-containing lipoproteins. An important advantage of the apoE^−/−^ model was an earlier publication that established the proatherogenic role of CETP in mice (29). Importantly, CETP expression in apoE^−/−^ mice did not enhance atherosclerosis by increasing circulating SAA, since SAA levels were generally lower, not higher, in mice administered AAV-CETP. Reduced plasma SAA levels in CETP-expressing mice were associated with significantly reduced hepatic expression of SAA1.1 and SAA2.1, consistent with an earlier published report showing increased hepatic SAA expression in CETP transgenic mice treated with a CETP inhibitor, anacetrapib (36). The mechanism by which CETP regulates hepatic SAA expression requires further investigation.

As noted previously, the vast majority of circulating SAA is normally associated with HDL, and one consequence of CETP remodeling is the dissociation of SAA from HDL. Thus, an alternative mechanism by which CETP reduces plasma SAA is through the generation of lipid-poor SAA, which may be deposited in tissues or susceptible to degradation. Immunohistochemistry staining revealed markedly increased amounts of SAA detected in aortic root sections of apoE^−/−^ mice expressing CETP, despite reduced amounts of circulating SAA. Whether the presence of increased SAA promotes the local production of IL-β in the environment of an atherosclerotic lesion needs to be determined; however, systemic inflammatory markers (IL6 and IL-1β) were not observed in apoE^−/−^ mice expressing CETP.

There is an emerging literature about the role of inflammation in CVD. Numerous studies have demonstrated correlations between inflammatory marker levels and CVD events, particularly not only for C-reactive protein (CRP) (40) but also for SAA (3, 4, 5, 6, 7). The CANTOS trial found that targeting IL-1β with canakinumab decreased recurrent CVD events, albeit at the cost of increased fatal infections (40). Furthermore, subgroup analyses suggested that patients whose CRP level dropped with canakinumab had the greatest benefit, suggesting that suppression of inflammation leads to the best outcomes. While the CIRT study did not show any benefit for the use of low-dose methotrexate on CVD outcomes, there was also no evidence that methotrexate lowered CRP, IL-1β, or IL-6 (41). Thus, while we acknowledge that CETP inhibition has not been found to decrease CVD events in most studies despite striking increases in HDL levels, it is possible that the majority of subjects in these studies did not have high levels of inflammation (42). Our data imply that CETP inhibition could be of use in prevention of CVD in individuals with chronic inflammation. In the setting of chronic inflammatory diseases with elevated SAA, the action of CETP to liberate SAA from HDL could unmask the proatherogenic activities of SAA, leading to the development of atherosclerosis. In these patients, CETP inhibition would minimize HDL remodeling, keeping SAA on HDL where its activity is largely sequestered, and minimize the proatherogenic effects of SAA. Further research would be needed to test this hypothesis, but based on our findings here, we argue there is validity to pursue this concept.

In summary, we conclude that the proatherogenic effect of CETP in apoE^−/−^ mice is mediated, at least in part, by SAA. Furthermore, our studies demonstrate that the known proatherogenic effect of SAA is increased by the expression of CETP, in part because of the action of CETP to liberate SAA from HDL. These novel findings suggest that CETP inhibition may be of particular therapeutic benefit in those individuals with high SAA levels.

## Data availability

Data will be shared upon request (University of Kentucky, preetha.shridas@uky.edu).

## Supplemental data

This article contains supplemental data.

## Conflict of interest

The authors declare that they have no conflicts of interest with the contents of this article.
