# Supplementary material for: Serum amyloid A augments the atherogenic effects of cholesteryl ester transfer protein
Source: J Lipid Res. 2023 Mar 31;64(5):100365. doi: 10.1016/j.jlr.2023.100365 (PMC10165456; doi:10.1016/j.jlr.2023.100365)
Supplement: Supplemental Fig. S1 [file mmc1.pdf]

Supplemental Figure S1

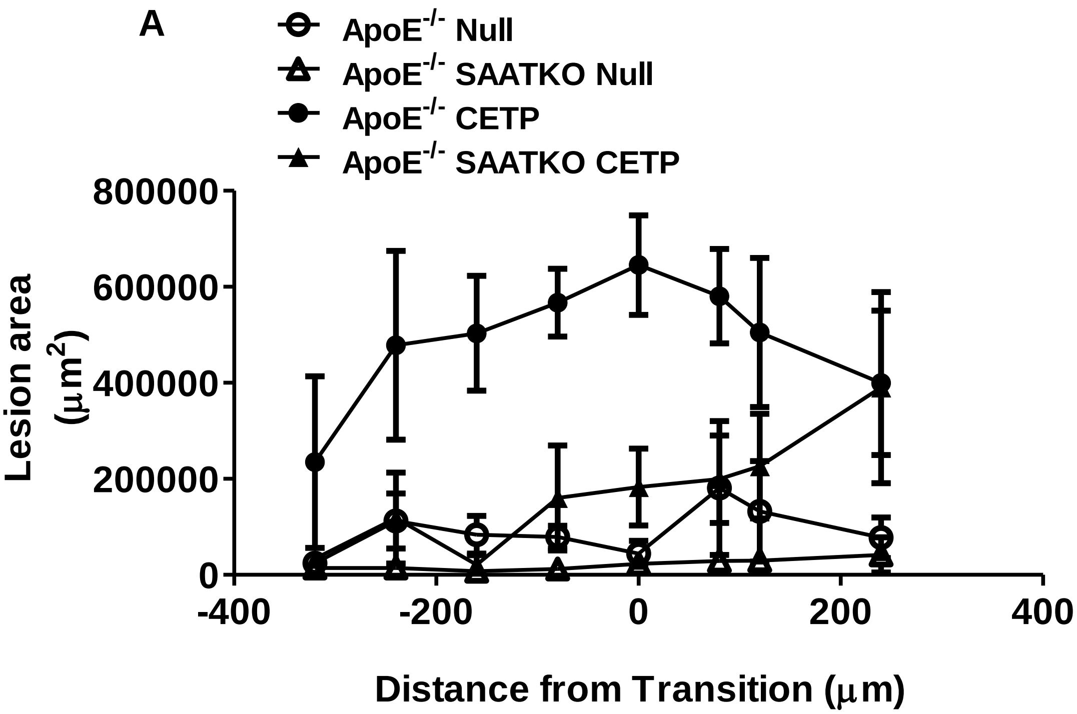

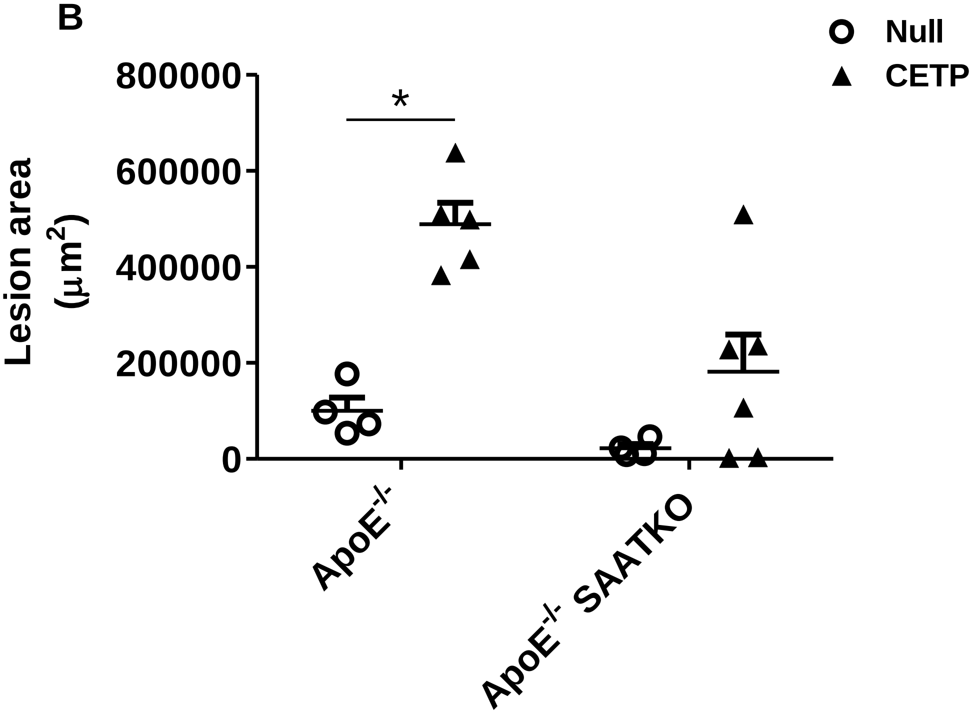

Supplemental Figure S1. **CETP expression increases lesion area in the roots of apoE$^{-/-}$ mice and not in apoE$^{-/-}$ SAATKO mice.** ApoE$^{-/-}$ and apoE$^{-/-}$ SAATKO mice were injected with an AAV expressing CETP or a null AAV and fed a normal chow diet for 14 weeks. **(A)** Atherosclerotic lesion area in aortic root sections were determined after oil red O staining. Circles represent the means and bars the SEM of sections located ~ 80 µm apart starting 320 µm before the transition and ending 240 µm after the transition. The transition zone was defined by the disappearance of the valve cusps and was labeled “0” on the x-axis; ApoE$^{-/-}$ Null (n = 4), ApoE$^{-/-}$ SAATKO Null (n = 4), ApoE$^{-/-}$ CETP (n = 5), ApoE$^{-/-}$ SAATKO CETP (n = 6). **(B)** Atherosclerotic lesion area in aortic root sections, values are the averages of 8 sections, covering a length of approximately 640 µm. Statistical analysis was performed using 2-way ANOVA followed by Tukey’s multiple comparisons test. ApoE$^{-/-}$ Null (n = 4), ApoE$^{-/-}$ SAATKO Null (n = 4), ApoE$^{-/-}$ CETP (n = 5), ApoE$^{-/-}$ SAATKO CETP (n = 6).
